# Shutter Speed Influences the Capability of a Low-Cost Multispectral Sensor to Estimate Turfgrass (*Cynodon dactylon L.*—Poaceae) Vegetation Vigor Under Different Solar Radiation Conditions

**DOI:** 10.3390/s26010047

**Published:** 2025-12-20

**Authors:** Rosa M. Martínez-Meroño, Pedro F. Freire-García, Nicola Furnitto, Sebastian Lupica, Salvatore Privitera, Giuseppe Sottosanti, Maria Spagnuolo, Luciano Caruso, Emanuele Cerruto, Sabina Failla, Domenico Longo, Giuseppe Manetto, Giampaolo Schillaci, Juan Miguel Ramírez-Cuesta

**Affiliations:** 1 Department of Ecology and Global Change, Desertification Research Centre (CIDE, CSIC-UV-GV), Moncada, 46113 Valencia, Spain; rm.martinez@csic.es (R.M.M.-M.); pedro.freire@csic.es (P.F.F.-G.); 2 Section of Mechanics and Mechanization, Department of Agriculture, Food and Environment (Di3A), University of Catania, 95123 Catania, Italy; nicola.furnitto@phd.unict.it (N.F.); sebastian.lupica@phd.unict.it (S.L.); salvatore.privitera1@unict.it (S.P.); giuseppe.sottosanti@unict.it (G.S.); maria.spagnuolo@unict.it (M.S.); or lcaruso@unict.it (L.C.); emanuele.cerruto@unict.it (E.C.); sabina.failla@unict.it (S.F.); or dlongo@unict.it (D.L.); giuseppe.manetto@unict.it (G.M.); giampaolo.schillaci@unict.it (G.S.)

**Keywords:** illumination, MAPIR Survey3, precision agriculture, radiometric correction, reflectance, vegetation indices

## Abstract

Radiometric calibration of multispectral imagery plays a critical role in the determination of vegetation-related features. This radiometric calibration strongly depends on a proper sensor configuration when acquiring images, the shutter speed being a critical parameter. The objective of the present study was to appraise the influence of shutter speed on the reflectance in the visible and near-infrared (NIR) spectral regions registered by a low-cost multispectral sensor (MAPIR Survey3) on a homogeneous field of turfgrass (*Cynodon dactylon L.*—Poaceae) and on the vegetation index (VI) values calculated from them, under different solar radiation conditions. For this purpose, 10 shutter speed configurations were tested in field campaigns with variable solar radiation values. The main results demonstrated that the reflectance in the green spectral region was more sensitive to shutter speed than that of the red and NIR spectral regions, particularly under high solar radiation conditions. Moreover, VIs calculated using the green band were more sensitive to slow shutter speeds, thus presenting a higher probability of providing meaningless artifact values. In conclusion, this study provides shutter speed recommendations under different illumination conditions to optimize the reflectance and the VI sensitivity within the image, which can be applied as a simple method to optimize image acquisition from unmanned aerial vehicles under varying solar radiation conditions.

## 1. Introduction

Nowadays, agriculture faces unprecedented challenges due to the effects of climate change and the rapid growth of the world’s population. Ensuring food security while meeting increasing demands requires strategies supported by accurate and timely crop monitoring [1]. Remote sensing technologies have emerged as essential tools for data-driven decision making and the optimization of field management strategies [2,3].

In this sense, recent advancements in multispectral and hyperspectral sensors have enabled the high-throughput, high-resolution monitoring of critical crop parameters [4,5]. These include the vegetation index (VI), leaf area index (LAI), and foliar chlorophyll concentration, which are key metrics for assessing plant physiological and bio-physical status [6,7]. These technologies facilitate non-destructive evaluations of plant responses to biotic and abiotic stressors, which can cause structural and biochemical changes, such as reductions in biomass and chlorophyll content [8,9].

Spectral reflectance (R_λ_) measurements and VIs are highly sensitive to several influencing elements. Factors such as canopy structure [10], illumination geometry [11], atmospheric conditions [12], and the biophysical characteristics of vegetation can introduce variability and inconsistencies in the recorded data [13]. In particular, radiation levels and camera settings, including shutter speed, play critical roles in determining the quality and reliability of R_λ_ measurements [14]. Specifically, shutter speed controls how long to keep the shutter open to capture an image before closing. Therefore, it affects the amount of light coming into the sensor. The faster the shutter speed, the less time light has to enter, whereas the slower the shutter speed, the more time light has to enter. If this parameter is not set properly, it can result in an underexposed image or in pixel saturation (the sensor receiving very little or too much light, respectively). Addressing these influences is essential for ensuring the consistency and accuracy of data acquired under varying environmental conditions.

In this context, low-cost multispectral sensors have garnered attention for their affordability and accessibility, making them attractive alternatives for precision agriculture applications [15]. These sensors offer a cost-effective alternative to high spatial and spectral resolution cameras, providing satisfactory results with a more affordable price point, a user-friendly interface, and enhanced practicality for integration into business applications [16]. Despite their potential, these sensors often face limitations related to sensitivity, calibration, and environmental adaptability [17]. A key challenge is understanding how variations in environmental factors, such as daily radiation levels, and operational settings, like shutter speed, affect the R_λ_ and VIs collected and calculated by multispectral sensors. This knowledge gap hampers the effective deployment of low-cost multispectral sensors, particularly in resource-limited settings, where maximizing their utility could be critical.

While high-end and advanced sensors have been thoroughly explored, low-cost multispectral sensors are increasingly recognized in environmental sciences for their ability to generate valuable monitoring data, enabling research in a wide range of fields and applications [18]. To our knowledge, current research has not examined in depth how changes in radiation levels and shutter speed impact the R_λ_ and VI values provided by low-cost sensors. This highlights the pressing need for studies that evaluate the performance of these accessible sensing technologies to enhance their reliability across diverse agricultural contexts.

In this framework, the hypothesis behind the present study is that an improper shutter speed configuration produces systematic distortions in the spectral bands acquired by a low-cost multispectral sensor, affecting the vegetation index values computed from them. Thus, the general objective of this study was to evaluate the feasibility of using a low-cost multispectral sensor to perform studies to estimate vegetation vigor. The specific objectives were as follows: (i) to assess the performance of the MAPIR Survey3 RGN multispectral camera under varying solar radiation levels and shutter speed settings using a leaf spectrometer as a comparative consistency check method; (ii) to study their influence on the R_λ_ of different bands measured by the sensor and on the VIs calculated from them; and (iii) to systematically analyze the relationship between shutter speed modulation and the R_λ_ collected by the multispectral sensor to identify critical transition points in the VI response.

## 2. Materials and Methods

### 2.1. Study Site and Meteorological Conditions

**Figure 1:**
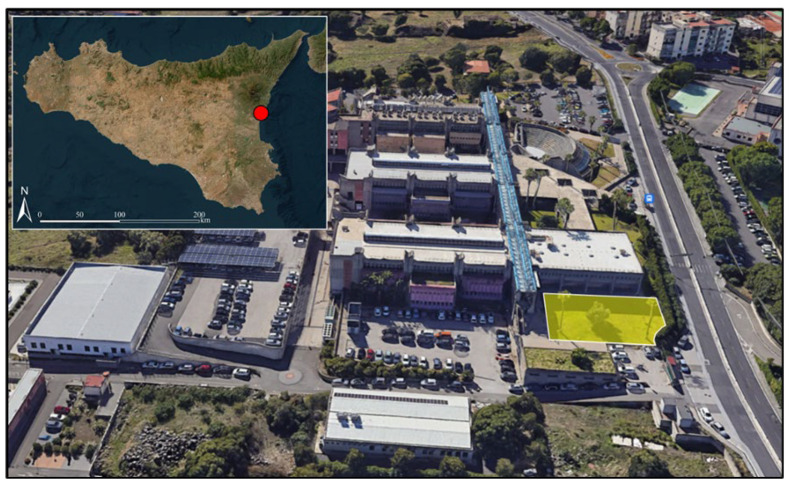
Location of the study site (red point and yellow polygon) within the facilities of the Department of Agriculture, Food and Environment of the University of Catania, Sicily, Italy (37.5364° N; 15.0679° E) (Credits: Esri, Maxar, Earthstar Geographics, and the GIS User Community; and Google Earth Pro on web version).

The study site (Figure 1) was a homogeneous field of turfgrass (*Cynodon dactylon L.*—Poaceae) located at the facilities of the Department of Agriculture, Food and Environment of the University of Catania (Sicily, Italy; 37.5364° N; 15.0679° E).

**Figure 2:**
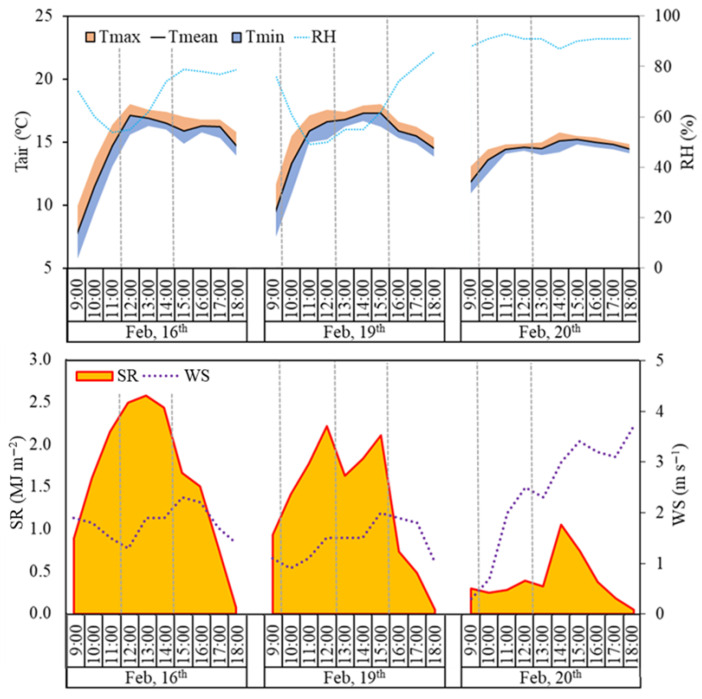
Meteorological data observed in the study site during the measurement campaigns (T_air_: air temperature; T_max_: maximum air temperature; T_mean_: mean air temperature; T_min_: minimum air temperature; RH: relative humidity; SR: solar radiation; WS: wind speed). Vertical dashed lines indicate the multispectral and hyperspectral data acquisition times.

A total of 7 measurement campaigns were carried out across 3 days that were representative of varying solar radiation conditions: (i) a totally sunny sky (February 16th 2024 with a mean solar radiation, SR, of 1.63 MJ m^−2^); (ii) intermittent cloud presence (February 19th with a SR value of 1.32 MJ m^−2^); and (iii) mostly cloudy conditions (February 20th with a SR value of 0.40 MJ m^−2^). These average SR values referred to the 9:00 to 18:00 period. Additionally, different moments of the day were covered in order to explore the intraday variations in solar radiation conditions. Figure 2 summarizes the meteorological conditions on the measurement campaign days at an hourly scale, including the air temperature (T_mean_), relative humidity (RH), SR, and wind speed (WS). Specifically, two measurements were taken on February 16th (11:30 and 14:30, local time) and February 20th (9:30 and 12:30, local time), while three measurements were acquired on February 19th (9:30, 12:30, and 15:30, local time). In detail, during the measurement period on February 16th and 19th, T_mean_ varied from around 7.7 °C to 17.3 °C, with RH values ranging from 50% to 83%. A more homogeneous behavior in terms of T_mean_ and RH was observed on February 20th, with T_mean_ varying from 11.8 °C to 15.2 °C and RH oscillating between 88% and 93%. WS was less than 2.5 m s^−1^ in all measurements. No precipitation occurred during the entire experiment. Hourly meteorological data were obtained from the Servizio Informativo Agrometeorologico Siciliano (http://www.sias.regione.sicilia.it/; accessed on March 15th 2025), specifically from the Catania weather station (37.4418° N; 15.0677° E) located ≈ 10 km from the study site.

### 2.2. Experimental Design

The measurement campaigns were performed on a randomly selected 1 m × 1 m region delimited within the turfgrass study area by using a handcrafted reference wooden frame. This structure was placed, when possible, under sunny conditions, avoiding the shadows of the multispectral sensor and surrounding elements (e.g., palm and olive trees and buildings). Within this area, multispectral and hyperspectral data were acquired.

#### 2.2.1. Multispectral Data

In each one of the measurement campaigns, nadiral multispectral data were acquired using a multispectral MAPIR Survey3 camera RGN model (MAPIR, San Diego, CA, USA). This sensor, which has a resolution of 4000 pixels × 3000 pixels, acquires 3 spectral bands located in the visible (red, 660 nm; and green, 550 nm) and near-infrared, NIR (850 nm) domains. A total of 10 multispectral images per campaign were acquired at different shutter speeds (S) selected from those available in the multispectral sensor (1 s, 1/8 s, 1/15 s, 1/30 s, 1/60 s, 1/90 s, 1/125 s, 1/250 s, 1/500 s and 1/1000 s), positioning the camera at 1 m above the wooden frame. Shutter speed values lower than 1/1000 s and higher than 1 s were discarded because, under the tested illumination conditions, the images were consistently under- or over-exposed, respectively. In order to assess only the effect of shutter speed configuration on the reflectance value acquired by the sensor, other camera parameters, such as the aperture (f/2.8), ISO/gain (ISO = 50), sensor linearity, or white balance, were kept invariant. Vignette (Flat Field) Correction was performed within MAPIR Camera Control (MCC) software to brighten the outer perimeter of pixels in the images, which appeared darker due to the lens optics.

This procedure was repeated across the seven measurement campaigns, resulting in a total of 70 images (7 campaigns × 10 images each). Details are provided in the Supplementary Material. Each measurement campaign, including all shutter speeds, took about 5–10 min.

The raw data were processed using the MAPIR Camera Control software, obtaining, for each considered shutter speed, the R_λ_ images in the red, green, and NIR bands. Before the image acquisition and for each shutter speed condition, 5 photographs were taken of a calibration panel, which needed to be included in the software for image pre-processing. This calibration panel contains 4 reference targets that have known reflectance (average R_λ_ values in the 550–850 domain are 0.87 for white target, 0.26 for light gray target, 0.22 for dark gray target, and 0.02 for black target). From them, the processing software creates reflectance calibration formulas to convert every pixel in an image into reflectance. These calibration formulas are specific to the current camera exposure settings, being affected by any exposure or ambient light change. This calibration procedure allows for comparison of the images with other calibrated data that may have been taken at different times of the day and from different locations around the world.

The variation in R_λ_ in each band as a function of the assessed S value was evaluated, selecting an S threshold corresponding to the maximum S value after which R_λ_ experienced a change of less than 0.04 with respect to the two subsequent lower S values. This value was empirically determined based on the observed R_λ_ behavior, and their generalization should be conditioned taking into account the site specificities (e.g., vegetation type or health status).

#### 2.2.2. Hyperspectral Data

**Figure 3:**
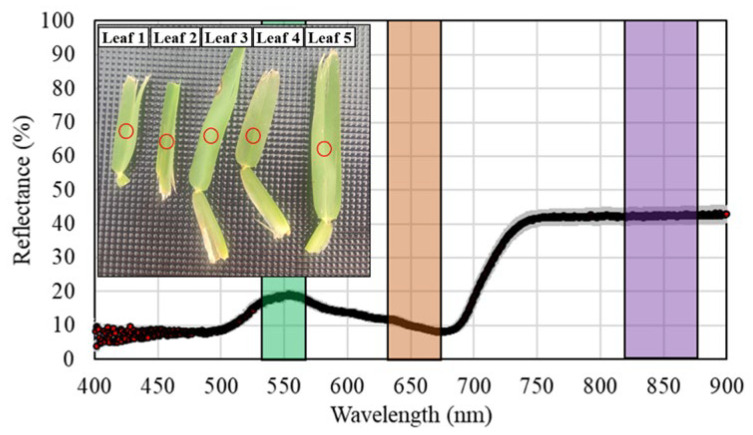
Leaf samples selected to obtain their visible and near-infrared hyperspectral signatures (gray area indicates the standard error). Red circles indicate the leaf area where the hyperspectral measurement was performed. Colored vertical areas in the graph identify the regions considered for averaging hyperspectral data for comparison purposes.

Simultaneously with each multispectral data acquisition, 5 healthy green leaves were regularly selected within the area covered by the wooden frame, and their spectral signature was obtained by using a CI-710s SpectraVue Leaf Spectrometer (CID Bio-Science Inc., Camas, WA, USA) (Figure 3). This sensor is a point spectrometer, composed of a Complementary metal–oxide–semiconductor (CMOS) Linear Array detector that measures transmission, absorption, and reflection on a range of wavelengths from 360 nm to 1100 nm, with a wavelength data width of 0.55 nm–0.70 nm.

For comparison purposes between the multispectral and hyperspectral data, the latter was averaged, taking into consideration the width of the spectral bands covered by the multispectral sensor (Figure 3). Specifically, hyperspectral data were averaged in the ranges of 543–558 nm, 653–668 nm, and 835–865 nm for the green, red, and NIR regions, according to the MAPIR Survey3 camera RGN model specifications.

#### 2.2.3. Vegetation Indices Calculation

From the R_λ_ values in the red (R_RED_), green (R_GREEN_), and NIR (R_NIR_), spectral bands obtained with the MAPIR camera in the 1 m × 1 m area with the different shutter speed configurations, five VIs (Equations (1)–(5)) were calculated using the MAPIR Camera Control software. Specifically, these indices included Normalized Difference Vegetation Index (NDVI; [19]), Optimized Soil Adjusted Vegetation Index (OSAVI; [20]), Chlorophyll Vegetation Index (CVI; [21]), Green Normalized Difference Vegetation Index (GNDVI; [22]) and Modified Simple Ratio (MSR; [23]).

(1) $$NDVI=\frac{R_{NIR}-R_{RED}}{R_{NIR}+R_{RED}},$$

(2) $$OSAVI=\frac{\left(R_{NIR}-R_{RED}\right)}{{(R}_{NIR}+R_{RED}+0.16)},$$

(3) $$CVI=\frac{R_{NIR}}{R_{GREEN}}\times \frac{R_{RED}}{R_{GREEN}},$$

(4) $$GNDVI=\frac{R_{NIR}-R_{GREEN}}{R_{NIR}+R_{GREEN}},$$

(5) $$MSR=\frac{\frac{R_{NIR}}{R_{RED}}-1}{\sqrt{\frac{R_{NIR}}{R_{RED}}}+1},$$

## 3. Results

### 3.1. Influence of the Shutter Speed on the Single Reflectance Bands

**Figure 4:**
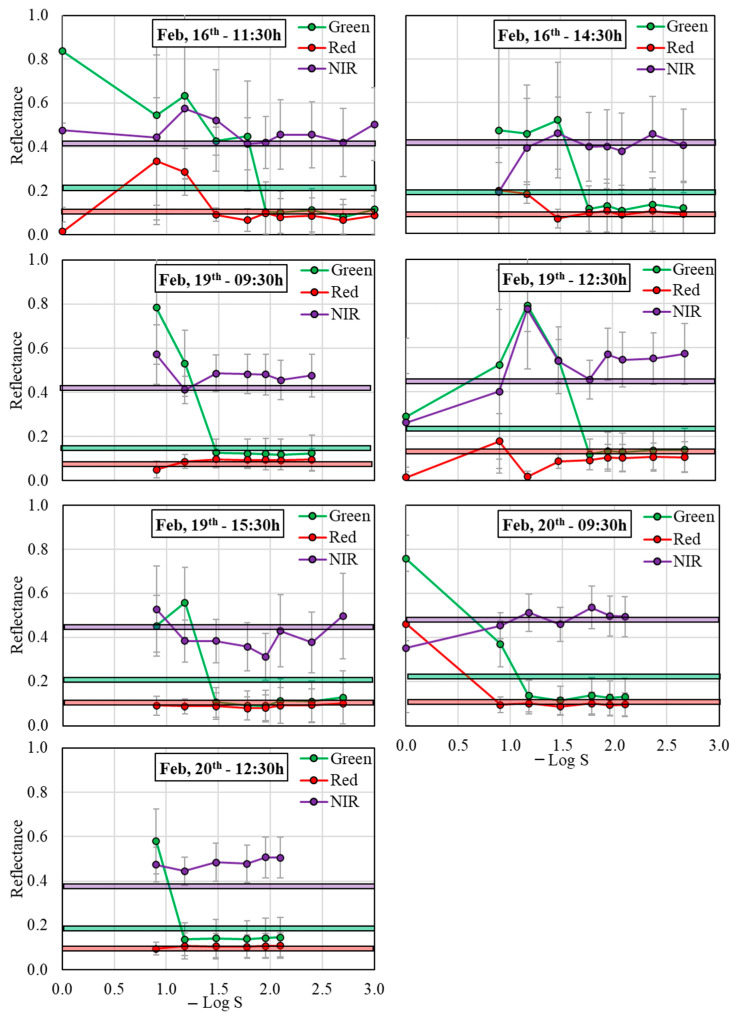
Reflectance values for the green, red, and near-infrared (NIR) bands obtained for each evaluated shutter speed and for the different acquisition times. Error bars represent the standard deviation. Horizontal lines reflect the average reflectance values obtained from the leaf spectrometer.

Figure 4 shows the R_λ_ values obtained from the MAPIR camera for the green, red, and NIR bands at the evaluated shutter speed configurations, and the R_λ_ values measured with the hyperspectral sensor. A general pattern was observed for all the measurement campaigns, presenting higher R_λ_ variability at high S values and reaching a near-constant R_λ_ value after a S threshold. This threshold was dependent on the solar radiation conditions during the measurement campaign and on the considered spectral band. Table 1 summarizes the S threshold recommendations as a function of the measured SR values.

Specifically, S threshold values for the green band were generally lower (higher −Log S in Figure 4) than for the red and NIR bands. In this sense, green band S threshold values varied between 1/90 s and 1/60 s (i.e., −Log S = 1.95 and 1.78, respectively) under sunny conditions (e.g., February 16th at 11:30 and 14:30), with R_GREEN_ values of 0.10–0.13 and a standard deviation (SD) for each S scenario lower than 0.15. For S configurations that were higher than the S threshold, R_GREEN_ reached values of 0.43–0.84 (SD = 0.06–0.41). Under mostly cloudy conditions (e.g., February 20th at 9:30 and 12:30), S threshold values varied from 1/8 s to1/15 s (i.e., –Log S = 0.90 and 1.18, respectively), with R_GREEN_ ranging from 0.12 to 0.15 and SD values lower than 0.09. For S configurations higher than the S threshold, under mostly cloudy conditions, R_GREEN_ reached values of 0.37–0.76 (SD = 0.10–0.37) (Figure 4).

For red and NIR bands, the S threshold values under sunny conditions were 1/30 s and 1/60 s, respectively (i.e., −Log S = 1.48 and 1.78, respectively) (e.g., February 16th at 11:30 and 14:30). For S configurations lower than the S threshold, R_RED_ and R_NIR_ varied from 0.07 to 0.10 and from 0.38 to 0.50, respectively, with SD values of 0.03–0.14 and 0.12–0.17, respectively, whereas for S configurations higher than the S threshold, R_RED_ and R_NIR_ varied from 0.01 to 0.34 and from 0.19 to 0.57, respectively, with SD values of 0.04–0.29 and 0.17–0.18, respectively. On the other hand, under mostly cloudy conditions (e.g., February 20th at 9:30 and 12:30), the S threshold reached a value of 1/8 s (i.e., −Log S = 0.90), with R_RED_ and R_NIR_ ranging from 0.09 to 0.11 and from 0.45 to 0.54, respectively, and SD values lower than 0.10. For S configurations higher than the S threshold, under mostly cloudy conditions, R_RED_ and R_NIR_ reached values of 0.46 (SD = 0.40) and 0.35 (SD = 0.35), respectively (Figure 4).

Moreover, some gaps were also evidenced in the R_λ_ values reported in Figure 4 at the extreme considered S conditions. For instance, they occurred at S = 1 s (i.e., −Log S = 0.00) on February 16th at 14:30; February 19th at 9:30 and 15:30; and on February 20th at 12:30; at S < 1/125 s (i.e., −Log S > 2.10) on February 20th; at S < 1/250 s (i.e., −Log S > 2.40) on February 19th at 9:30; and at S = 1/1000 s (i.e., −Log S = 3.00) on February 16th at 14:30, February 19th at 12:30 and 15:30 (Figure 4).

**Figure 5:**
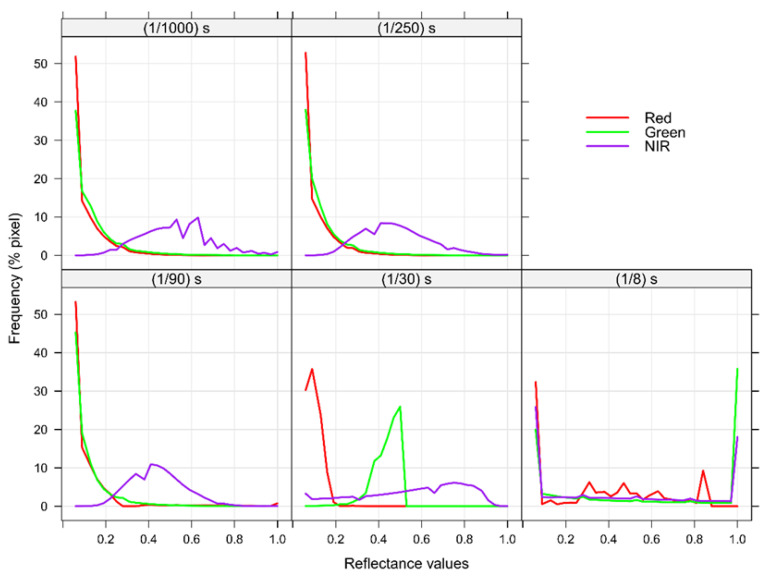
Example of frequency polygons obtained for the green, red, and NIR spectral bands of the processed images acquired at five shutter speed configurations on February 16th—11:30 (n = 573,227 pixels).

The frequency of R_λ_ values within the wooden frame polygon corroborated these patterns (Figure 5). Generally, at slow S configurations (e.g., 1/8 s; Figure 5), pixel distribution was concentrated at the extreme R_λ_ values (i.e., 0 and ≈ 1) in all evaluated spectral bands, whereas the frequency of pixels at intermediate R_λ_ values was almost negligible. As shutter speed became faster, the pixel distribution oscillated (e.g., S = 1/30 s in Figure 5) until reaching an S configuration where the polygon became almost invariant to S changes (e.g., S = 1/90 s and S = 1/250 s in Figure 5). Contrarily, at very fast S configurations (i.e., S = 1/1000 s), less light was integrated, and more pixels reached a minimal close-to-zero reflection value (Figure 5).

**Figure 6:**
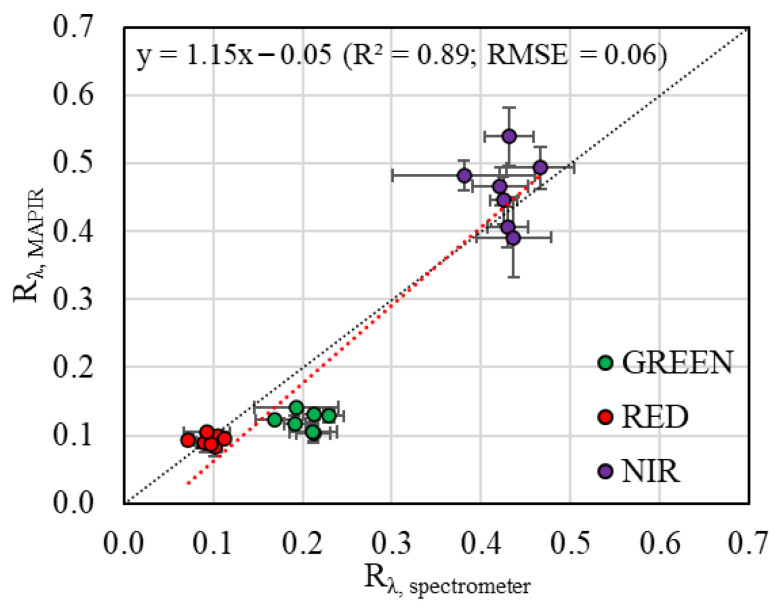
Relationship between the reflectance values measured with the leaf spectrometer (R_λ,spectrometer_) and with the MAPIR sensor (R_λ,MAPIR_). Error bars represent the standard deviation. Dashed black line represents the 1:1 relationship.

The comparison between the R_λ_ values obtained from the MAPIR camera after reaching the near-constant behavior with the spectral signature derived from the leaf spectrometer revealed a close relationship between them (R^2^ of 0.89 and RMSE = 0.06). This relationship was more accurate in the spectral domain of the red and NIR regions, with RMSE values of 0.01 and 0.06, respectively (Figure 6), whereas higher discrepancies were observed in the green spectral region, with the R_GREEN_ values derived from the multispectral sensor being lower than the values registered with the leaf spectrometer, resulting in a higher RMSE value (0.08; Figure 6).

### 3.2. Influence of the Shutter Speed on Vegetation Indices Calculation

**Figure 7:**
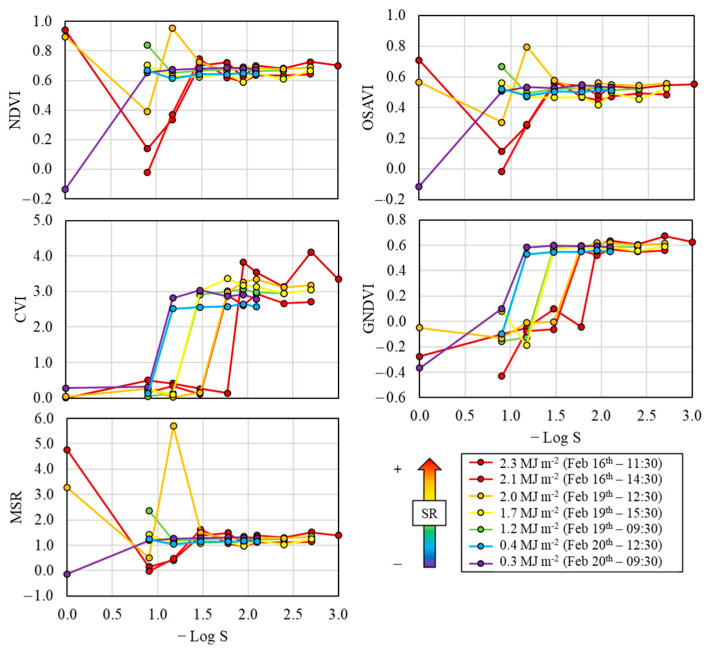
Vegetation index values calculated for each field campaign at the different shutter speed configurations.

The influence of shutter speed on the different VIs calculated from the R_λ_ values registered by the multispectral sensor is shown in Figure 7. As happened for the individual multispectral bands, it can be observed that all VIs reached a constant behavior at a certain S threshold value. In this sense, these S threshold values were dependent on the solar radiation and on the considered VIs. Generally, the S value threshold was lower (higher −Log S in Figure 7) as the solar radiation increased. Regarding the specific VIs, NDVI, OSAVI, CVI and MSR exhibited S threshold values that ranged from S = 1/15 s (−Log S = 1.18) under mostly cloudy conditions (e.g., February 20th at 9:30 and 12:30) to S = 1/90 s (−Log S = 1.95) under sunny conditions (e.g., February 16th at 11:30 and 14:30), whereas the S threshold values for GNDVI varied from S = 1/8 s (−Log S = 0.90) under mostly cloudy conditions (e.g., February 20th at 9:30 and 12:30) to S = 1/30 s (−Log S = 1.48) under sunny conditions (e.g., February 16th at 11:30 and 14:30). Thus, in terms of NDVI, OSAVI, CVI and MSR, the S values range suitable (i.e., constant pattern for all the measurements) for all radiation levels analyzed was from S = 1/90 s to S = 1/125 s (−Log S = 1.95 and 2.10, respectively), whereas this S range was wider for GNDVI, from S = 1/30 s to S = 1/125 s (−Log S = 1.48 and 2.10, respectively) (Figure 7).

## 4. Discussion

One of the main limitations when working with low-cost multispectral sensors is that they do not perform the shutter speed calibration automatically, and their respective post-processing software is typically unable to process images acquired with different shutter speeds. However, setting this parameter properly is critical since it determines the amount of light the camera receives, thus critically influencing the quality of the image [24]. Thus, the user must carefully set the value of this parameter in order to obtain valid images. However, these sensors are generally intended to be used by farmers or technicians, who rarely have the necessary knowledge to set this parameter, which could result in an underuse or incorrect use of the sensor.

For the specific sensor used in the present study (i.e., MAPIR Survey3 RGN model), the manufacturer provides general and qualitative default exposures recommendations differentiating between “Sunny to Light Cloudy Day” (S = 1/250 s) and “Medium Cloudy Day” (1/125 s) conditions (www.mapir.camera). These S recommendations were slightly faster than the band-dependent S threshold values identified in our study both under sunny (from 1/60 s to 1/90 s for the green band and from 1/30 s to 1/60 s for the red and NIR bands) and mostly cloudy conditions (from 1/8 s to 1/15 s for the green band and 1/8 s for the red and NIR bands). The application of generalist S values slower than the S threshold values determined experimentally led to a lower range of digital numbers (i.e., there is a range of the upper digital numbers that is not covered in the image) and consequently to a lower R_λ_ sensitivity. Thus, the use of the solar radiation-based S values contributes to an optimization of the R_λ_ sensitivity (i.e., the smallest recognizable R_λ_ difference), enhancing the discrimination of pixels with different R_λ_ values within the image. This concept acquires special relevance when coping with applications that deal with small R_λ_ variations, such as the classification of different crops [25,26,27]. Moreover, when the sensor was set with extreme S conditions (e.g., 1 s under sunny conditions and 1/1000 s under mostly cloudy conditions), the sensor was not able to apply the spectral correction due to the impossibility of detecting the calibration panel.

When considering the S thresholds reported in the present study, the R_λ_ values for the different spectral bands derived from the MAPIR showed a close agreement with the values measured with the leaf spectrometer, except for the green band, where the R_GREEN_ values derived with the MAPIR were lower than those provided by the leaf spectrometer. Such discrepancies could be due to the status of the vegetation measured with each sensor. Thus, while MAPIR measured a 1 m × 1 m area containing a mixture of green and senescent vegetation, the measures with the leaf spectrometer were performed on green leaves, thus presenting higher R_GREEN_ values. Thus, further investigation is required to confirm such a hypothesis. MAPIR Survey 3 sensor limitations, such as the noise and its spectral distortion, can also influence the R_λ_ value discrepancies found when compared to the hyperspectral sensor. In this sense, uncorrected sensor limitations, such as the noise or the potential non-linearity, could have introduced systematic and random errors that may have affected the findings derived from the present study. Specifically, sensor noise can lead to increased measurement uncertainty (signal-to-noise ratio decrease) since noise makes it harder to detect true signal changes [28]. The measurement quality was also subjected to errors caused by non-linearity, which introduced a systematic error that could be especially relevant in some specific cases, such as high-accuracy measurements or relative measurements between two or more wavelengths [29]. Additionally, the size of the sampled area does not allow ecological/agronomic generalization, since it could not represent the inherent spatial variability typical of real agricultural applications.

Moreover, the S thresholds determined in the present study were found to be band-dependent. In this sense, the green band was more sensitive to shutter speed than the red and NIR bands, especially at high solar radiation conditions. This means that, regardless of solar radiation conditions, the green band was the first to become overexposed as shutter speed increased (i.e., as −Log S decreased), due to the fact that maximum irradiance in the solar spectrum occurs at green wavelengths [30,31,32].

The influence of illumination conditions on several VIs has been evaluated in previous studies [33,34,35], but generally, such evaluation has pre-imposed a constant shutter speed. However, the main novelty of the present study is that it deepened on the variation in VIs with shutter speed under different solar radiation conditions. Thus, the band dependence of the response of R_λ_ to shutter speed was also observed in terms of VIs. In this sense, those VIs which used the green band for its calculation (e.g., CVI and GNDVI) were more sensitive to slow shutter speeds (i.e., more likely to saturate [36]). Thus, it would be advisable to use VIs based on other bands, such as the red or the NIR, that are less sensitive to solar radiation conditions (e.g., NDVI, OSAVI or MSR), allowing to set the sensor within a wider range of S values (S from 1/30 s to 1/500 s in comparison to S values from 1/90 s to 1/500 s, in the case of using green-based VIs).

Several studies have addressed the application of radiometric correction methods to multispectral images acquired from UAVs under varying illumination conditions [37,38,39,40,41]. Due to the capacity of UAVs for flying below the clouds, the acquired images could be exposed to varying meteorological conditions (e.g., solar radiation or illumination conditions), thus reducing the reliability of the generated products [33,42].

This problem was not as evident when working with satellite data due to the different areas covered by both platform types. For instance, the average covered by a single Landsat 9 scene is ≈33,000 km^2^ [43] versus an example of maximum area coverage of less than 200 km^2^ found by [44]. Specifically, most local and regional studies based on satellite data required a single scene, whereas the use of UAVs, more intended for local studies, required the mosaicking of several scenes, thus being susceptible to illumination changes between different acquisitions. Such illumination variations between single image acquisitions generate artifacts in the final orthomosaics, thus influencing the individual spectral bands and subsequent image-derived products, such as VIs [45].

Finally, further research is required to evaluate the effects of jointly setting various camera parameters such as the sensor aperture, ISO/gain, sensor linearity, or white balance.

## 5. Conclusions

The results derived from the present study evidenced the critical role that multispectral sensor configuration and, in particular, the shutter speed, had on the reflectance collected by the sensor at the different spectral bands and on the vegetation indices calculated from them on a homogeneous field of turfgrass. This study revealed that a proper calibration of the low-cost camera allowed it to reach reflectance values comparable to those achievable by the ones provided by more sophisticated sensors (e.g., hyperspectral cameras). Specifically, the main conclusions can be summarized as follows:

- The general thresholds recommended for setting shutter speed varied from 1/15 s at low solar radiation conditions (SR ≤ 0.4 MJ m^−2^) to 1/90 s under high solar radiation conditions (SR > 2.1 MJ m^−2^);
- The reflectance in the green spectral region was more sensitive to shutter speed than that of the red and NIR spectral regions, especially at high solar radiation conditions;
- Vegetation indices using the green band in their calculation were more sensitive to slow shutter speeds, thus presenting a higher probability of providing meaningless artifact values;
- A slight saturation of the green spectral band can result in an increase in the reflectance in the red and NIR bands, and of the non-green-based vegetation index sensitivity, enhancing the discrimination of pixels with different values within the image.

The research findings underscore the critical importance of accurate spectral data analysis and interpretation in agricultural applications. Moreover, the results offer an operational guidance for farmers or technicians for an optimal shutter speed adjustment as a function of the solar radiation at the image acquisition time (Table 1). However, the generalizability of the results from the present study to other regions is limited by the temporal scope of data collection and the small and unique geographical area covered. Based on these insights, future research should focus on key areas not deeply addressed in the present study, such as (i) the investigation of automated calibration techniques for optimizing shutter speed parameters under dynamic illumination conditions, potentially incorporating machine learning algorithms to enhance sensor response adaptation; (ii) the development of standardized protocols for spectral data acquisition using low-cost multispectral sensors across different temporal and environmental conditions, particularly focusing on the relationship between exposure time and spectral reflectance stability; (iii) a comprehensive analysis of the impact of varying shutter speeds on specific vegetation indices across different phenological stages and stress conditions, with emphasis on establishing reliable measurement thresholds; and (iv) the extension of the current methodology to larger areas, diverse vegetation types and environmental conditions to validate the robustness of the identified critical transition points in spectral response.

## Figures and Tables

**Table 1:** Shutter speed thresholds identified for the MAPIR Survey3 camera RGN model as a function of the measured solar radiation under the conditions and vegetation type tested.

Solar Radiation (MJ m^−2^)	Shutter Speed Threshold (s)
SR ≤ 0.4	1/15
0.4 < SR ≤ 1.7	1/30
1.7 < SR ≤ 2.1	1/60
SR > 2.1	1/90

## Data Availability

The raw data supporting the conclusions of this article will be made available by the authors on request.

## Supplementary Materials

The following supporting information can be downloaded at: https://www.mdpi.com/article/10.3390/s26010047/s1, Figure S1: Example of raw and processed images acquired at five shutter speed configurations (1/1 s,1/8 s, 1/15 s, 1/30 s and 1/60 s) on February 16th—11:30.; Figure S2: Example of raw and processed images acquired at five shutter speed configurations (1/90 s, 1/125 s, 1/250 s, 1/500 s and 1/1000 s) on February 16th—11:30.
