# Supplementary material for: Shutter Speed Influences the Capability of a Low-Cost Multispectral Sensor to Estimate Turfgrass (Cynodon dactylon L.—Poaceae) Vegetation Vigor Under Different Solar Radiation Conditions
Source: Sensors (Basel). 2025 Dec 20;26(1):47. doi: 10.3390/s26010047 (PMC12788143; doi:10.3390/s26010047)
Supplement: Supplementary file 1 [file sensors-26-00047-s001.zip › sensors-3990597-supplementary.pdf]

Article

Shutter Speed Influences the Capability of a Low-Cost Multispectral Sensor to Estimate Turfgrass (*Cynodon dactylon L.*—Poaceae) Vegetation Vigor Under Different Solar Radiation Conditions

Rosa M. Martínez-Meroño [1], Pedro F. Freire-García [1], Nicola Furnitto [2], Sebastian Lupica [2], Salvatore Privitera [2], Giuseppe Sottosanti [2], Maria Spagnuolo [2], Luciano Caruso [2], Emanuele Cerruto [2], Sabina Failla [2], Domenico Longo [2], Giuseppe Manetto [2], Giampaolo Schillaci [2] and Juan Miguel Ramírez-Cuesta [1,*]

[1] Department of Ecology and Global Change, Desertification Research Centre (CIDE, CSIC-UV-GV), Moncada, 46113 Valencia, Spain; rm.martinez@csic.es (R.M.M.-M.); pedro.freire@csic.es (P.F.F.-G.)

[2] Section of Mechanics and Mechanization, Department of Agriculture, Food and Environment (Di3A), University of Catania, 95123 Catania, Italy; nicola.furnitto@phd.unict.it (N.F.); sebastian.lupica@phd.unict.it (S.L.); salvatore.privitera1@unict.it (S.P.); giuseppe.sottosanti@unict.it (G.S.); maria.spagnuolo@unict.it (M.S.); luciano.caruso@unict.it or lcaruso@unict.it (L.C.); emanuele.cerruto@unict.it (E.C.); sabina.failla@unict.it (S.F.); domenico.longo@unict.it or dlongo@unict.it (D.L.); giuseppe.manetto@unict.it (G.M.); giampaolo.schillaci@unict.it (G.S.)

* Correspondence: ramirezcuesta.jm@gmail.com; Tel.: +34-699-689-673

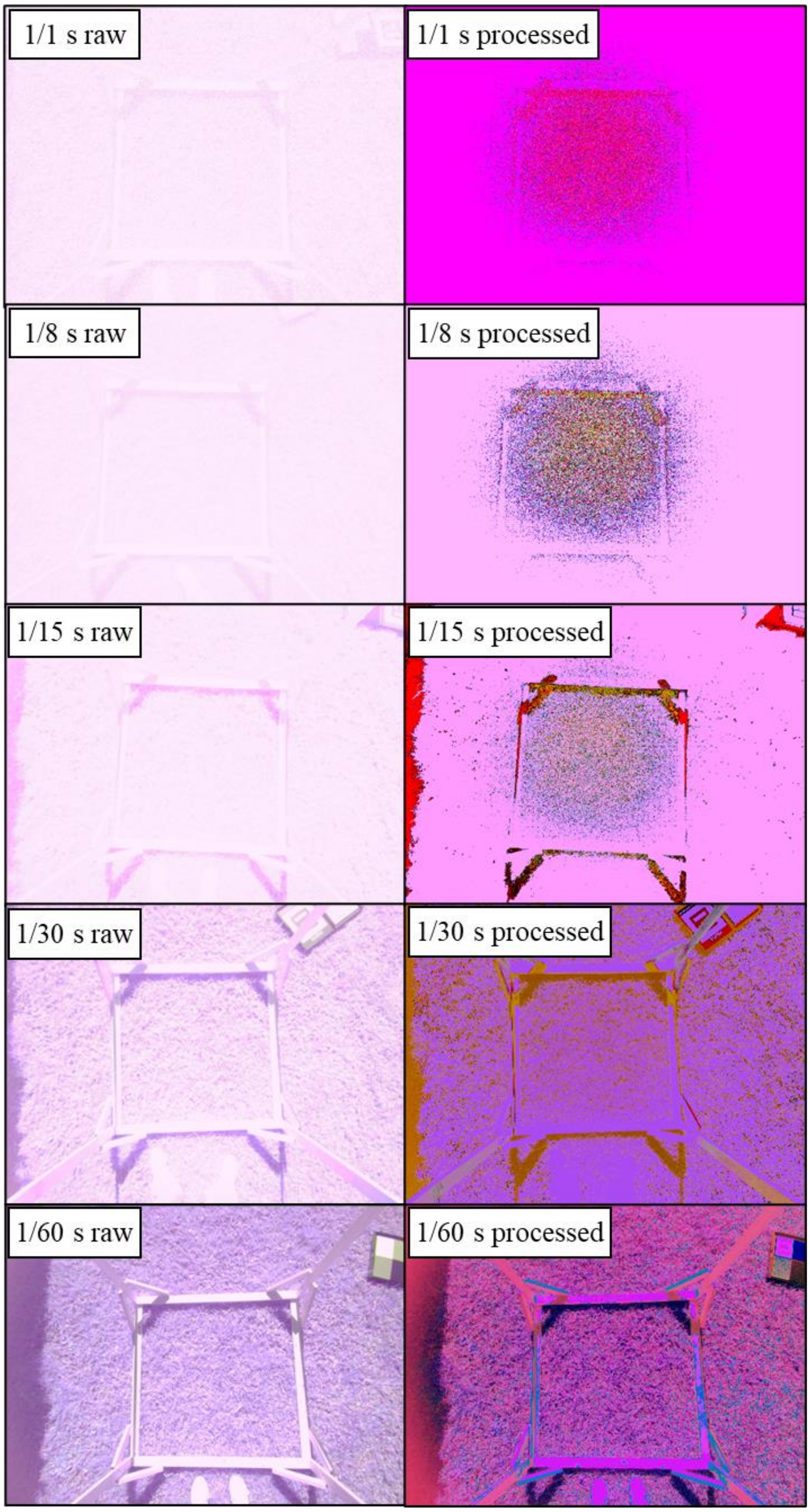

Figure S1. Example of raw and processed images acquired at five shutter speed configurations (1/1 s,1/8 s, 1/15 s, 1/30 s and 1/60 s) on February 16th - 11:30.

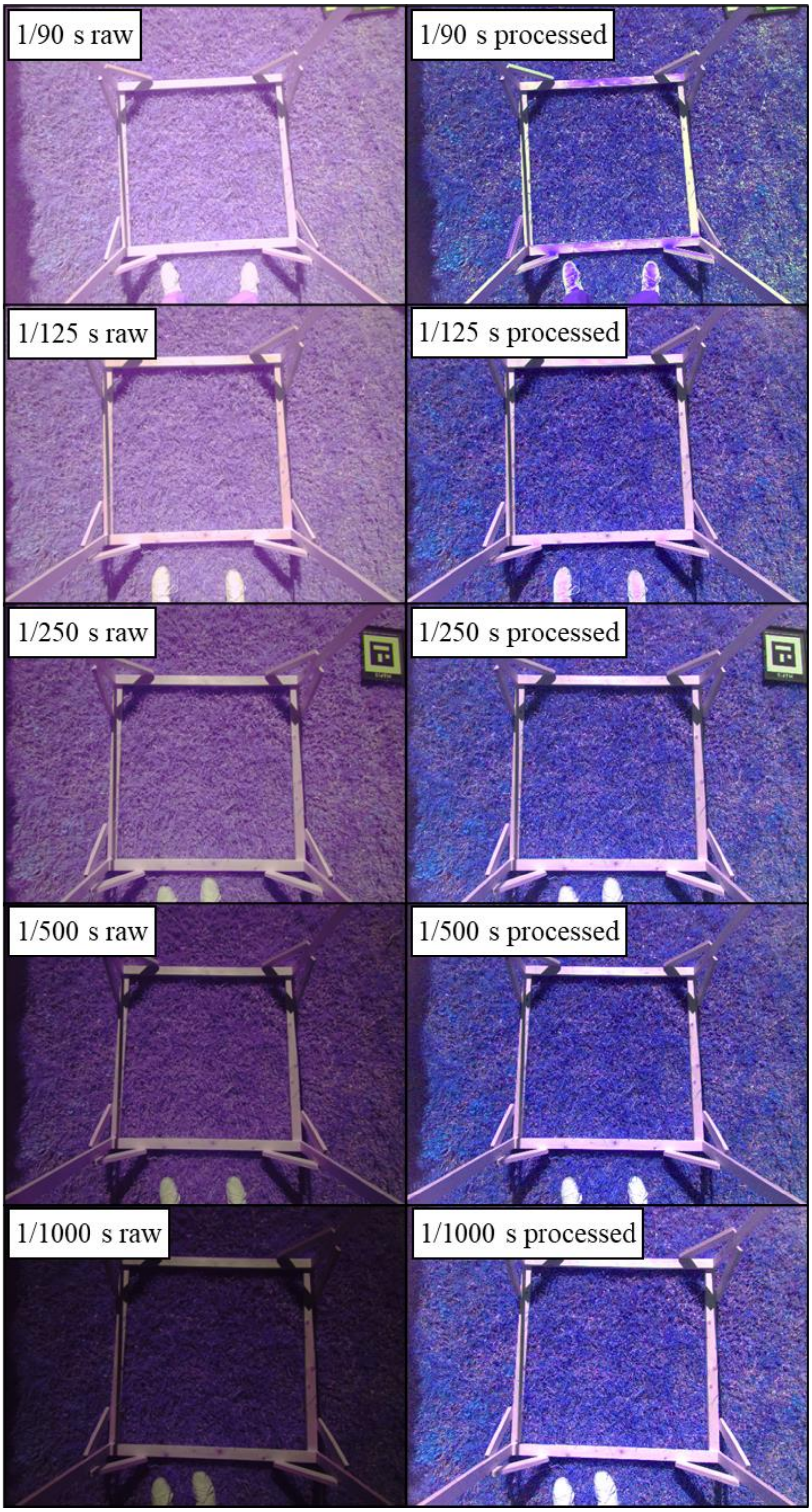

Figure S2. Example of raw and processed images acquired at five shutter speed configurations (1/90 s, 1/125 s, 1/250 s, 1/500 s and 1/1000 s) on February 16th - 11:30.
